# Role of Prox1 in the Transforming Ascending Thin Limb of Henle's Loop during Mouse Kidney Development

**DOI:** 10.1371/journal.pone.0127429

**Published:** 2015-05-19

**Authors:** Yu-mi Kim, Wan-Young Kim, Sun Ah Nam, A-Rum Choi, Hyang Kim, Yong-Kyun Kim, Hak-Soo Kim, Jin Kim

**Affiliations:** 1 Department of Anatomy and Cell Death Disease Research Center, College of Medicine, The Catholic University of Korea, Seoul, Korea; 2 Division of Nephrology, Kangbuk Samsung Hospital, Sungkyunkwan University, School of Medicine, Seoul, Korea; 3 Department of Internal Medicine, College of Medicine, The Catholic University of Korea, Seoul, Korea; Emory University, UNITED STATES

## Abstract

The homeobox transcription factor Prox1 is critical to the development of many embryonic organs and tissues, although current understanding of its expression in the developing renal medulla is limited. We examined the functional role of Prox1 during mouse kidney development with particular emphasis on the developing loop of Henle. Our data show that Prox1 is expressed in the transdifferentiating region from the NKCC2-positive thick ascending limb, into the CLC-K1-positive ascending thin limb of Henle’s loop beginning at embryonic day 18. From 1 to 14 days of age, Prox1-positive cells gradually disappeared from the papillary tip, and remained in the initial part of inner medulla after 21 days. In this transforming area, no Prox1 was observed in cells undergoing apoptosis but was expressed strongly in the remaining cells, which differentiated into ascending thin limb epithelial cells. *In vitro* and *in vivo* approaches showed that Prox1 expression increases where the osmolality is near optimal range, but decreases at below- or above-optimal ranges. Renal hypoosmolality induced by furosemide (NKCC2 inhibitor) inhibited Prox1 expression and delayed maturation of the ascending limb of Henle’s loop. Together, these studies suggest that Prox1 appears to be a critical stage specific regulator of specifying ascending thin limb cell fate and that its expression is regulated by osmolality.

## Introduction

The mammalian kidney contains short-looped and long-looped nephrons. The thin limb segments of the long loop of Henle maintain a hypertonic medullary interstitium and mediate formation of concentrated urine by the countercurrent multiplier system of the renal medulla [1]. The neonatal mammalian kidney (380–800 mOsm/kg) does not concentrate urine to the same extent as the adult kidney does (1200–1400 mOsm/kg). The diminished ability to concentrate urine is thought to be due to immaturity of tubular structure and function [2]. During the initial stages of kidney development, the inner medulla is not yet formed. All loops of Henle are short loops without an ascending thin limb (ATL), until immediately before birth. We previously reported that the cuboidal epithelium of the thick ascending limb (TAL) is removed by apoptosis and the surviving cells are converted into the squamous cells of the ATL during the first 2 weeks after birth. In the postnatal period, the inner-outer medullary border forms as a result of maturation of Henle’s loop via transformation of the ascending limb (AL) in the renal papilla [3,4]. We also showed that expression of the Na^+^-K^+^-2Cl^-^ cotransporter (NKCC2), tonicity-responsive enhancer binding protein (TonEBP), aldose reductase (AR), and urea transporter mediate urine concentration and maturation of the renal papilla in rats and mice. These findings suggest a relationship between maturation of the AL of Henle’s loop and osmolality via an unknown mechanism [5–8].

The homeobox gene *prox1*, homologue of the *Drosophila* gene *prospero*, encodes a transcription factor with a prospero domain and a homeodomain [9–13]. Prox1 is an essential regulator in the formation of a variety of organs and cell types, including the lymphatic vascular system, lens, retina, liver, pancreas, and heart [14–23].

A recent study in our laboratory demonstrated Prox1 expression during lymphatic vascular development in the mouse kidney. In all LYVE-1-positive lymphatic vessels of the renal cortex, Prox1 appeared in the fetus at 13 days (F13) and continued into adulthood [24]. Interestingly, Prox1 was also expressed in the renal papilla and expression levels varied with maturation of Henle’s loop.

In this study, we show that Prox1 expression contributed to maturation of the AL of Henle’s loop in the adult and developing mouse kidneys. We used *in vitro* and *in vivo* approaches in adults to show that Prox1 responds to osmolality. Furthermore, by reducing osmolality in developing mouse kidney, we show that Prox1 expression is related to maturation of the AL of Henle’s loop. Thus, our study demonstrates the importance of Prox1 in the developing mouse kidney and maturation of the AL of Henle’s loop.

## Results

### Prox1 expression in the adult mouse kidney

**Fig 1:**
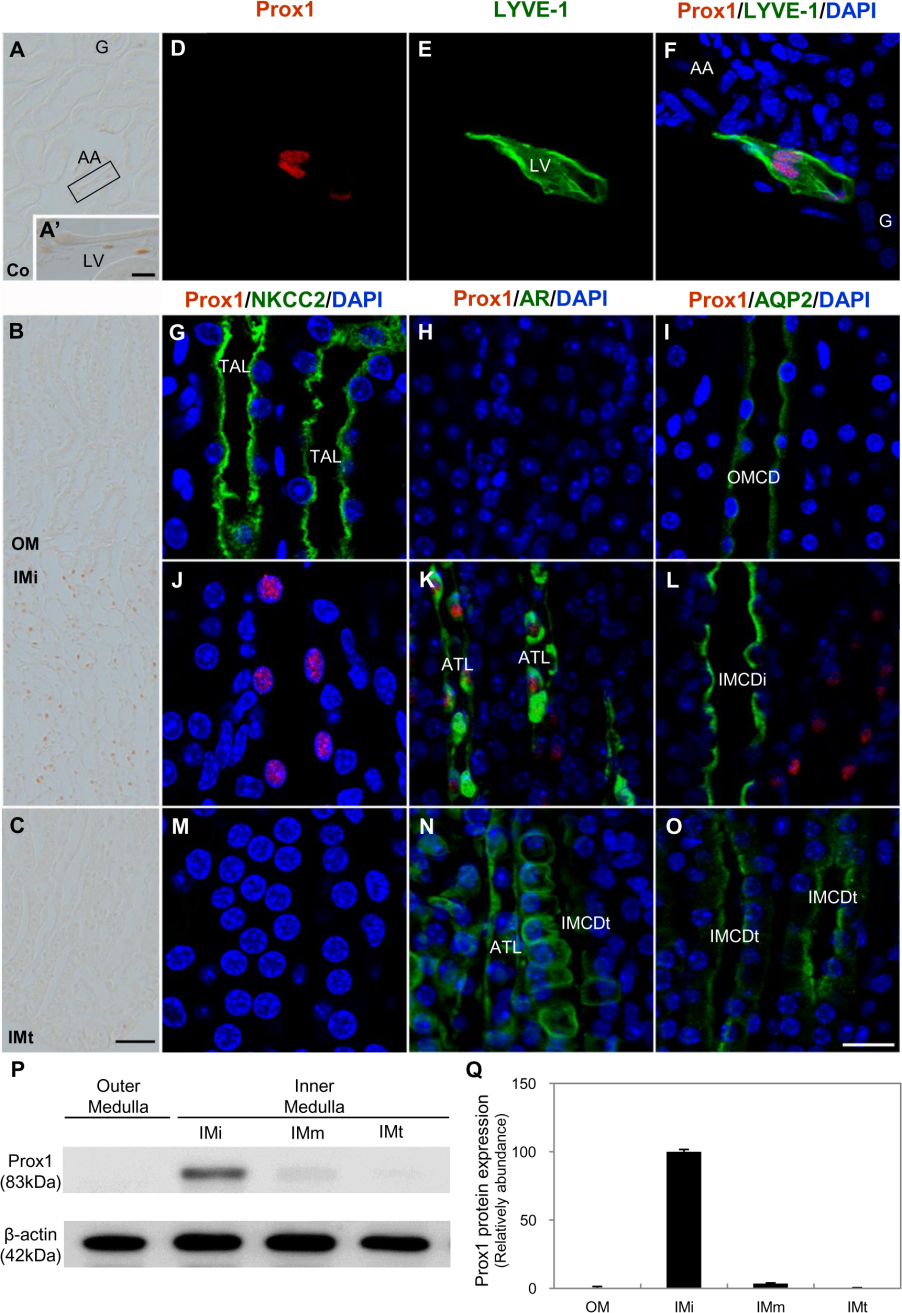
Expression of Prox1 in the adult mouse kidney. (A-C) Single immunostaining for Prox1. Scale bars: 200 μm. Co, cortex; G, glomerulus; AA, arcuate artery; LV, lymphatic vessel. (A’) Inset: higer magnification view of rectangle in A. Scale bars: 10 μm. (D–O) Double immunofluorescence staining for Prox1 (red) and LYVE-1 (green), Prox1 (red) and NKCC2 (green), Prox1 (red) and AR (green), and Prox1 (red) and AQP2 (green). Blue counterstain: DAPI. Scale bars: 20 μm. OMCD, outer medullary collecting duct; IMCDi, initial inner medullary collecting duct; IMCDt, terminal inner medullary collecting duct. (P–Q) Western blot analysis of Prox1 in the outer medulla (OM), and initial (IMi), middle (IMm), and terminal (IMt) part of the inner medulla. Band intensity was normalized to β-actin. Each bar is the mean of three independent experiments; error lines are SD.

Prox1 was expressed mainly in the nuclei of LYVE-1-positive lymphatic endothelial cells in the mouse renal cortex (Fig 1A and 1D–1F) [24]; it was also expressed in the renal medulla. Prox1 was expressed only in the initial part of the inner medulla, but not in the outer medulla or the middle and terminal parts of the inner medulla (Fig 1P and 1Q). Multiple-probe immunohistochemistry confirmed Prox1 localization in the initial part of the inner medulla. Prox1 was expressed in the AR-positive ATL in the initial part of the inner medulla, especially near the inner-outer medullary border, but not in the middle and terminal parts of the inner medulla. No expression was observed in the NKCC2-positive TAL in the outer medulla, or in AQP2-positive CD throughout the renal medulla (Fig 1B, 1C and 1G–1O).

### Prox1 expression in the developing renal papilla

**Fig 2:**
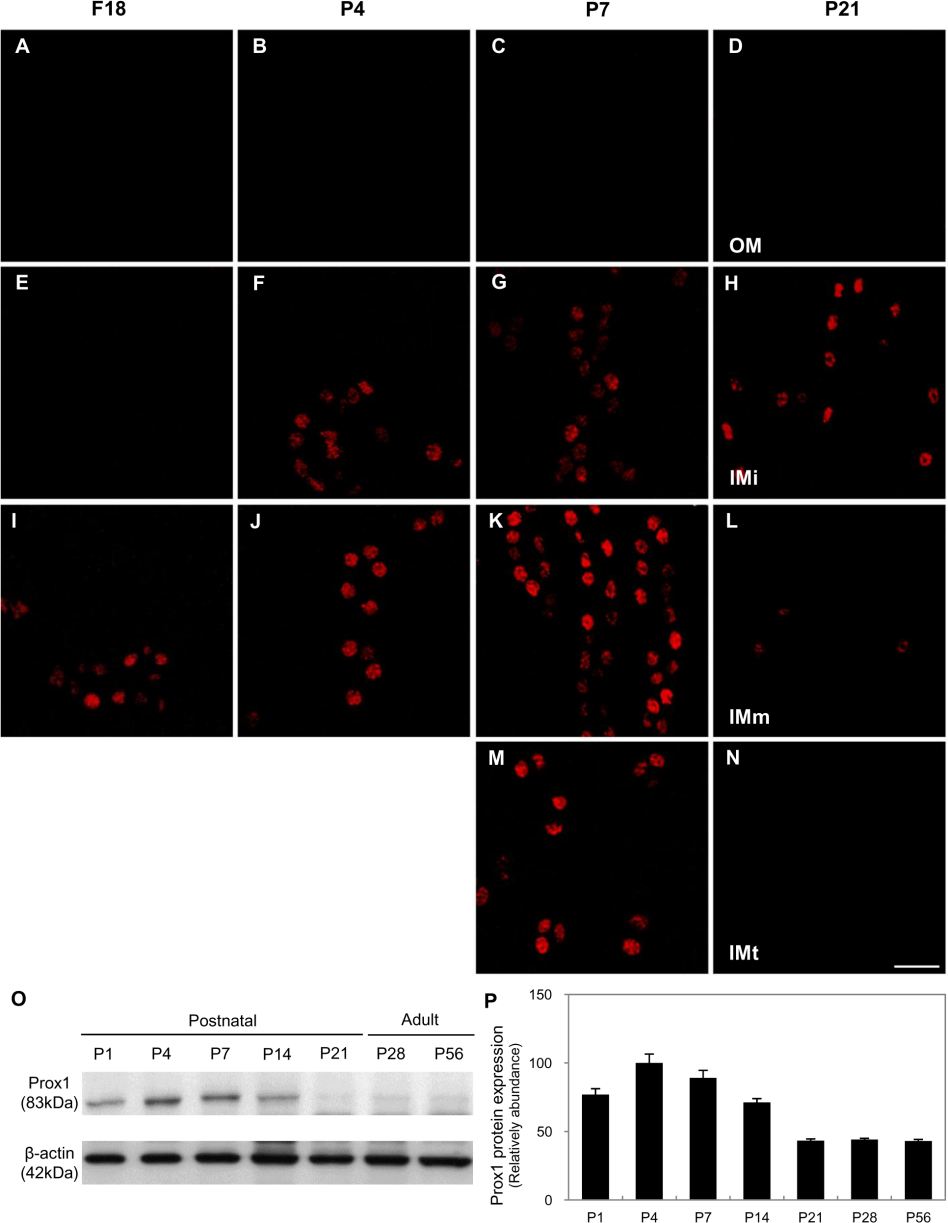
Prox1 expression during mouse kidney development. (A-N) Immunofluorescence staining of Prox1 (red) in the kidneys from 18-day-old fetuses (A, E, I) and from 4- (B, F, J), 7- (C, G, K, M) and 21-day-old pups (D, H, L, N). Blue counterstain: DAPI. Scale bars: 20 μm. (O-P) Western blot analysis of Prox1 in the mouse renal medulla at postnatal days 1, 4, 7, 14, 21, 28, and 56. Band intensity was normalized to β-actin. Each bar is the mean of three independent experiments; error lines are SD.

We demonstrated that cuboidal epithelial cells in the TAL are removed by apoptosis and the surviving cells are converted into the squamous cells of the ATL during the first 2 weeks after birth in rats (S1 and S2 Figs) [4]. Prox1 appeared first near the tip of the renal papilla on F18 and gradually increased thereafter. After birth, Prox1 expression gradually disappeared from the tip of the renal papilla until 21-day-old pups (P21), after which it remained only in the initial part of the inner medulla (Fig 2A–2N). Prox1 protein levels gradually increased until P7, and then decreased from P14 to adulthood (Fig 2O–2P).

To establish the precise sites of Prox1 expression in the transforming loop of Henle, TALs and ATLs were identified by immunolabeling with antibodies to NKCC2 and kidney-specific chloride channel (CLC-K1), respectively. We also identified descending thin limbs (DTLs; AQP1), CD (AQP2), and vasa recta (VR; CD31).

**Fig 3:**
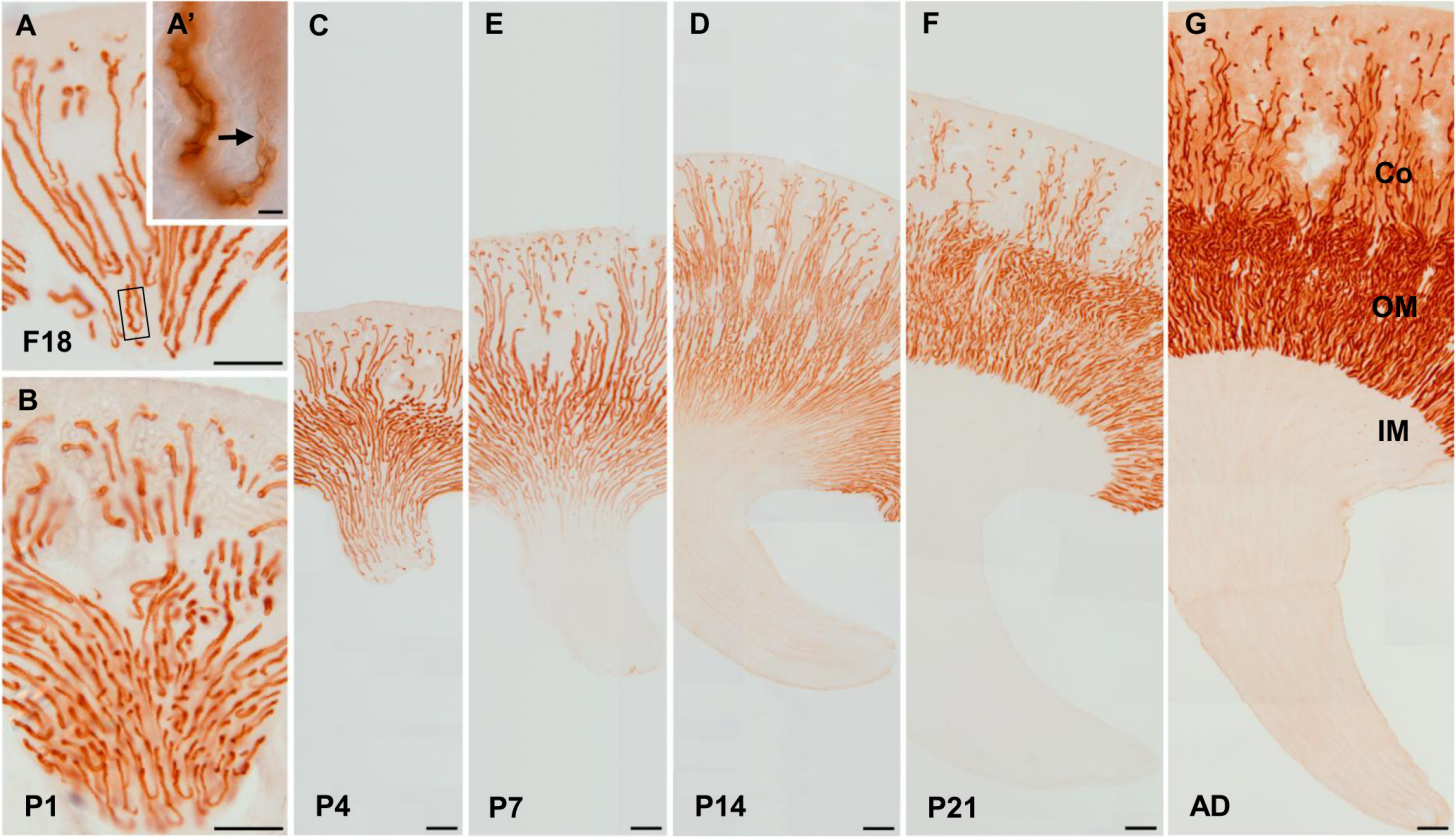
NKCC2 expression during mouse kidney development. Immunostaining of NKCC2 in the kidneys from 18-day-old fetuses (A) and from 1- (B), 4- (C), 7- (D), 14- (E) and 21-day-old pups (F) and adults (G). Scale bars: 200 μm. (A’) Inset: higer magnification view of rectangle in A, demonstrated that the descending thin limb (arrow) continues directly into the NKCC2-positive thick ascending limb. Scale bars: 10 μm.

At F18, cuboidal NKCC2-labeled TALs were located throughout the renal papilla, some reaching the tip (Fig 3A). They were directly connected to the poorly differentiated DTLs just before the bend of the loop (Fig 3A). At this stage of development, there were no CLC-K1-positive ATLs, and all loops of Henle had only TALs which were composed of simple cuboidal epithelium.

The number of NKCC2-positive TALs decreased in the lower portion of the renal papilla throughout the period of 1–14 days after birth (Fig 3B–3E). The disappearance of NKCC2 immunostaining started at the papillary tip and ascended until almost disappearing from the inner papilla at P7; however, NKCC2-positive TALs remained in the mid and outer portions (Fig 3D). In the kidneys of 14-day-old pups, the NKCC2-positive TALs remained only in the outermost part of the renal papilla, close to the outer medulla (Fig 3E). In contrast, squamous CLC-K1-positive ATLs appeared at the papillary tip and ascended throughout the stages of development. Interestingly, the lower cuboidal epithelial cells located between the NKCC2-positive TALs and CLC-K1-positive ATLs stained positive for both NKCC2 and CLC-K1.

NKCC2-positive TALs were no longer observed in the inner medulla of P21 (Fig 3F). Thus, there was a well-defined boundary between the outer and inner medulla, marked by the abrupt transition from the CLC-K1-positive ATLs to the NKCC2-positive TALs in adult animals. The NKCC2/CLC-K1-positive transitional portion was not observed in pups beyond 21 days.

**Fig 4:**
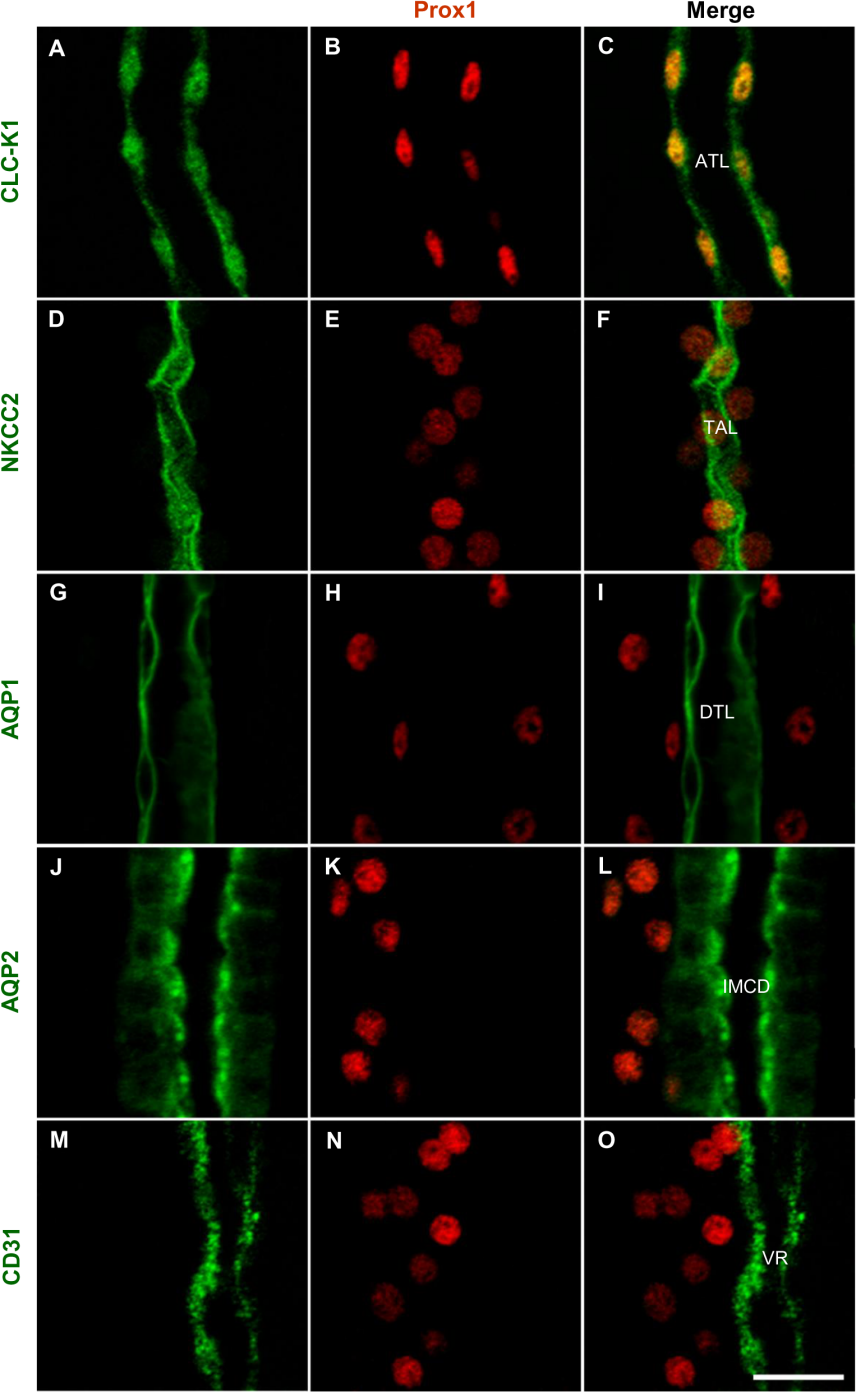
Prox1 expression in the developing mouse kidney. Double immunofluorescence staining for CLC-K1 (A, green) and Prox1 (B, red), NKCC2 (D, green) and Prox1 (E, red), AQP1 (G, green) and Prox1 (H, red), AQP2 (J, green) and Prox1 (K, red), and CD31 (M, green) and Prox1 (N, red) in renal papilla of 4-day-old pups. Prox1 was co-expressed in CLC-K1-positive ATL (C) or NKCC2-positive TAL (F), but not in AQP1-positive DTL (I), AQP2-positive CD (L), and CD31-positive VR (O). Scale bars: 20 μm

Prox1 was expressed in CLC-K1-positive ATL and/or NKCC2-positive TAL, but not in AQP1-positive DTL, AQP2-positive CD, or CD31-positive VR (Fig 4).

**Fig 5:**
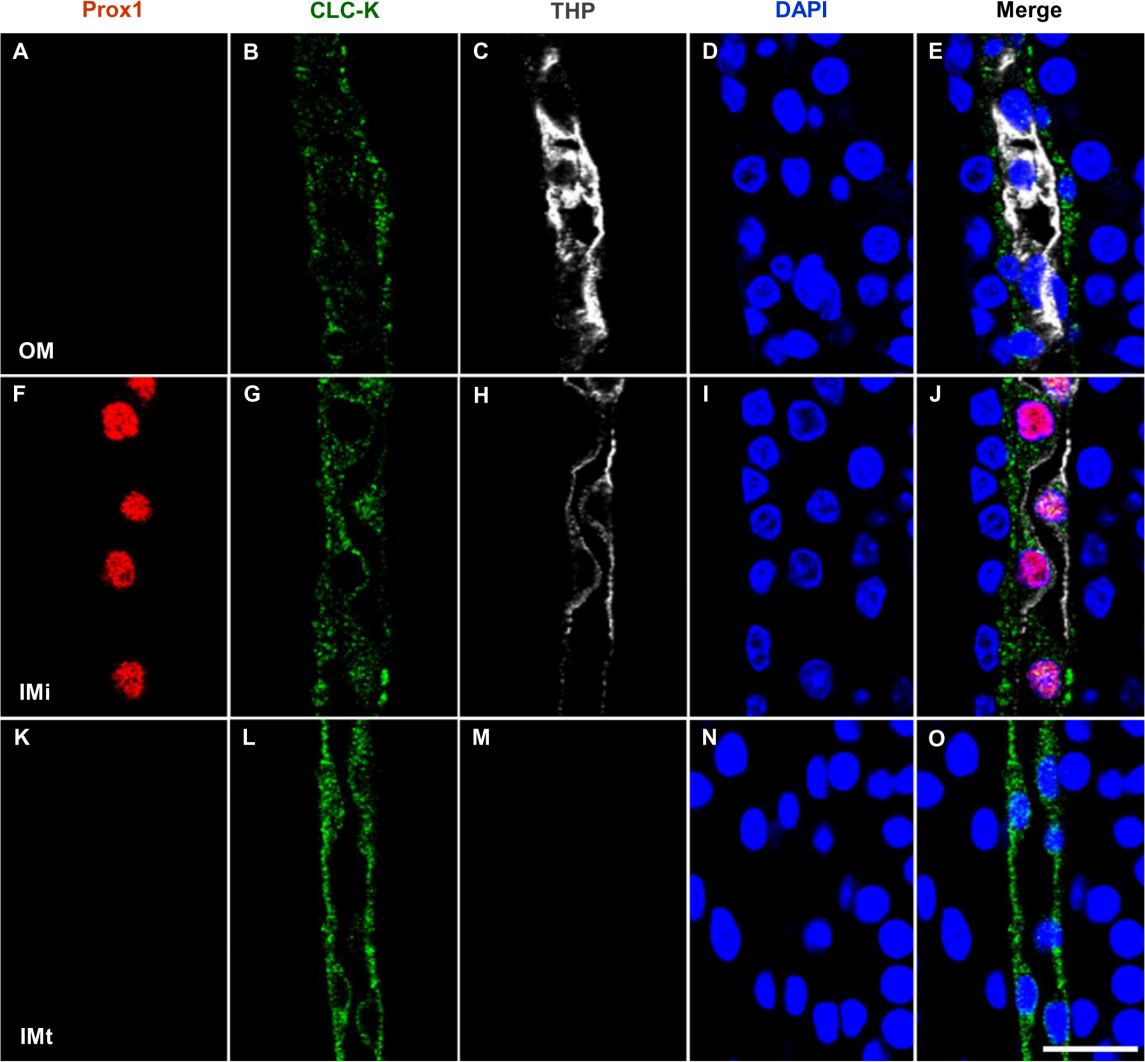
Prox1 during maturation of the AL of Henle’s loop in mouse kidney development. Triple immunofluorescence staining of Prox1 (red), CLC-K1 (green), and THP (white) in the renal papilla of 14-day-old pups. Prox1 was observed in the transforming region from TAL to ATL (F–J) and was not expressed in the mature TAL (A–E) or mature ATL (K–O). Blue counterstain: DAPI. Scale bars: 20 μm

In the developing loop of Henle, Prox1 expression appeared first at the bend of the NKCC2-positive TALs at the tip of the renal papilla in F18 (S3 Fig). From P1 to P14, Prox1 was observed in the CLC-K1-positive ATLs and in the transforming region (Fig 5F–5J). However, Prox1 was not expressed in the mature TAL (Fig 5A–5E) or ATL (Fig 5K–5O). Prox1-positive nuclei were observed only in the CLC-K1-positive ATLs of the initial part of the inner medulla in the kidneys of 21-day-old pups. No Prox1 immunoreactivity was observed in the NKCC2-positive TALs in the outer medulla or in CLC-K1-positive ATLs in the middle and terminal inner medulla beyond 21 days.

### Correlation between Prox1 expression and apoptosis or proliferation

**Fig 6:**
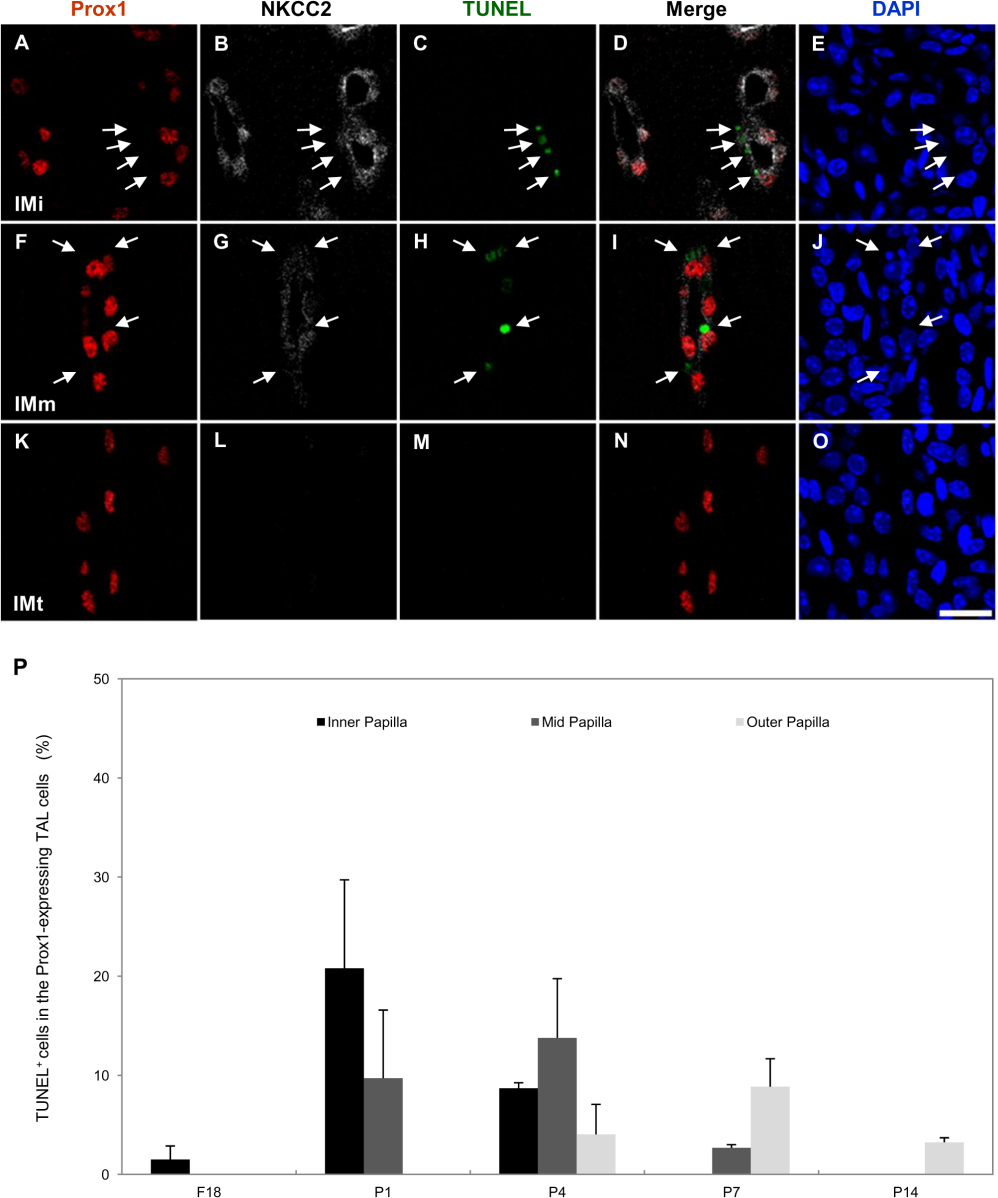
Cells undergoing apoptosis in the Prox1- and NKCC2-positive TAL of the developing mouse kidney. (A-O) Triple immunofluorescence staining for Prox1 (red), NKCC2 (white) and TUNEL (green) in the renal papilla from 7-day-old pups. Arrows indicate TUNEL-positive nuclei that did not co-localize with Prox1. Blue counterstain: DAPI. Scale bars: 20 μm. (P) Cells undergoing apoptosis are expressed as a percentage of the Prox1-positive TAL cells. Values are means ± SD.

**Fig 7:**
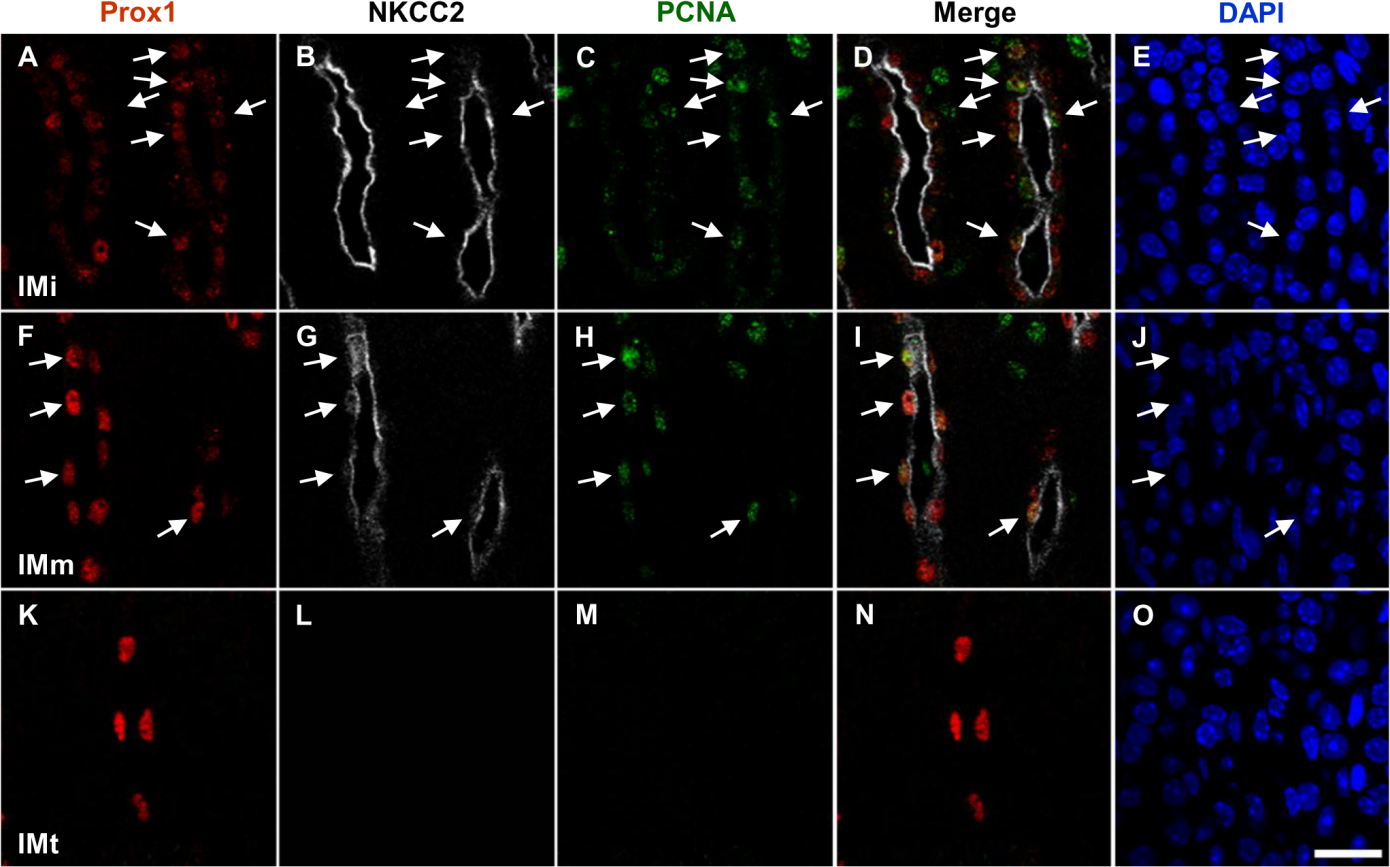
Co-localization of Prox1 and PCNA in the NKCC2-positive TAL of the developing mouse kidney. Triple immunofluorescence staining for Prox1 (red), NKCC2 (white) and PCNA (green) in the renal papilla from 7-day-old pups. PCNA co-localized with Prox1 signals in the nuclei of TAL cells. Blue counterstain: DAPI. Scale bars: 20 μm.

Immature TALs in the renal papilla are transformed into ATLs by apoptotic deletion and transformation of the remaining cells into a thin squamous epithelium [4]. To evaluate whether the observed colocalization between Prox1 and apoptotic cells or remaining cells, we performed double immunohistochemistry with Prox1 and terminal deoxynucleotidyl transferase dUTP nick-end labeling (TUNEL) or proliferating nuclear cell antigen (PCNA). Apoptotic cells appeared first in the Prox1-positive tubules in the inner papilla right after birth, and the apoptotic wave passed through the midportion and the outer portion of the papilla by P7. TUNEL-positive nuclei did not co-localize with Prox1 (Fig 6). PCNA-positive nuclei were mainly observed in the TAL cells, and only a few were observed in segments undergoing transformation or in newly formed ATL cells. Some of the Prox1-positive cells in the transforming AL co-localized with PCNA (Fig 7).

### Correlation between Prox1 expression and urine osmolality

**Fig 8:**
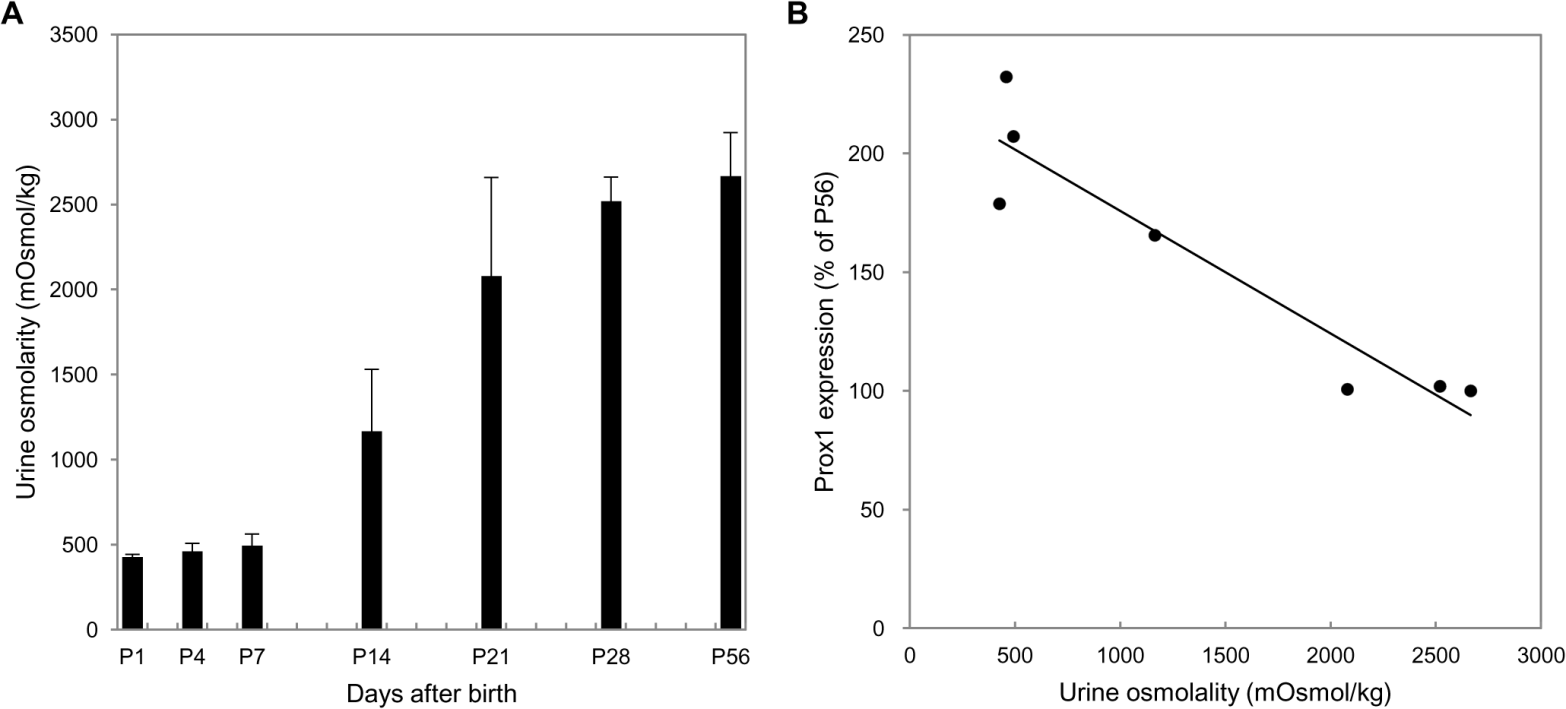
Osmolality and Prox1 expression. (A) Urine osmolalities were measured in mice at postnatal days 1, 4, 7, 14, 21, 28, and 56 on *ad libitum* fluid intake. Within 1 week after birth, urine osmolality was no more than 500 mOsmol/kg H_2_O; it began to increase from day 14, reaching peak levels at days 28 and 56. Each bar represents the mean of three independent experiments; error lines are SD. (B) At all developmental stages, Prox1 expression correlated closely with urine osmolality. R^2^ = 0.8870; P = 0.0015

Within 1 week after birth, the urine osmolality was no more than 500 mOsmol/kg H2O. At P14, urine osmolality was slightly higher but remained lower than in adult mice. It increased rapidly from P21 and was significantly higher by P28 and P56 (adults) (2520 ± 141 and 2666 ± 257 mOsmol/kg H2O, respectively; Fig 8A). A positive correlation was seen between expression of Prox1 in renal medulla according to developmental age and urine osmolality with *ad libitum* fluid intake (R^2^ = 0.8870, P = 0.0015, Fig 8B).

### Expression of Prox1 in response to changes in osmolality

**Fig 9:**
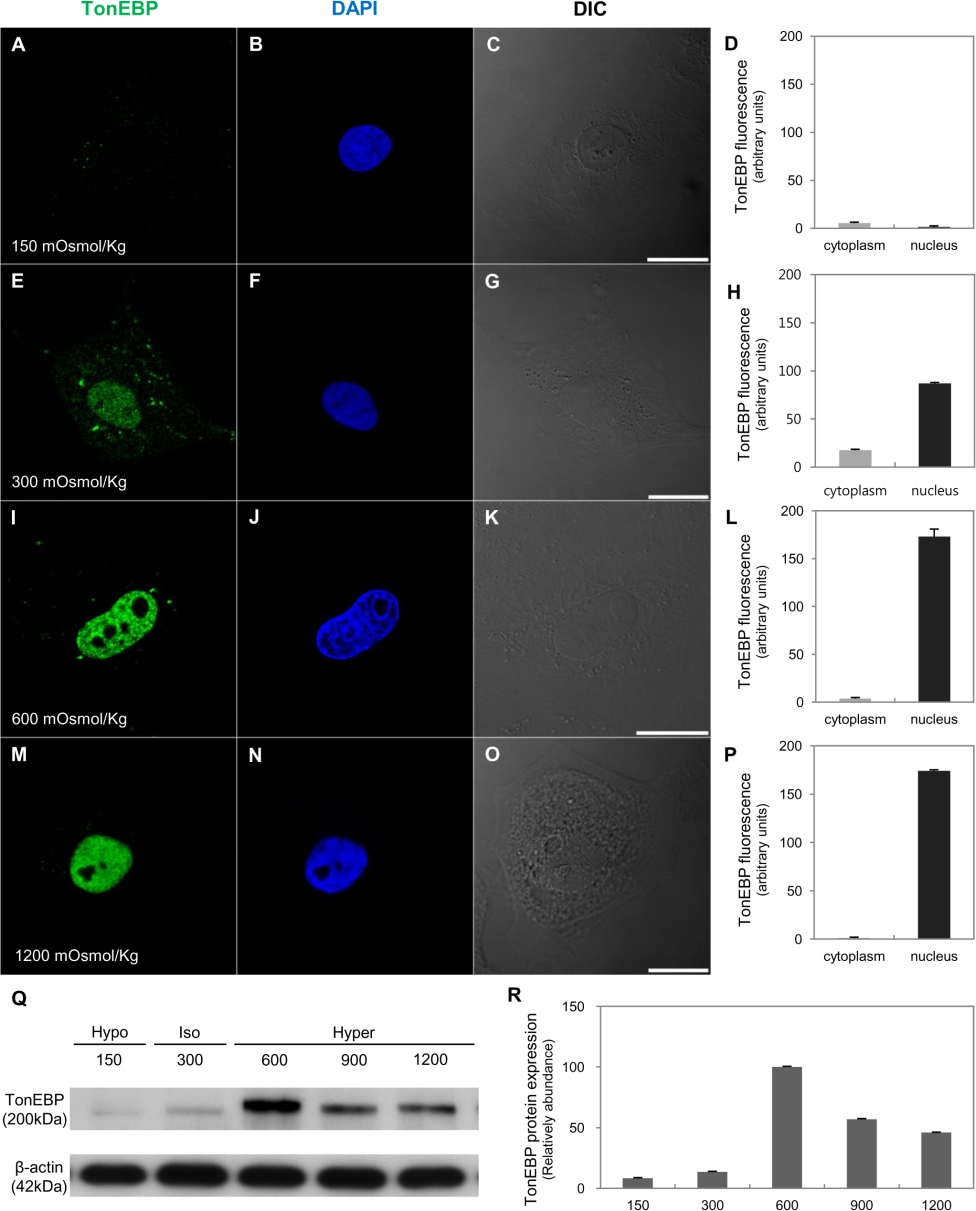
Expression and distribution of TonEBP in response to changes in osmolality *in vitro*. Immunofluorescence staining of TonEBP (green) in MDCK cells from 150 (A–D), 300 (E–H), 600 (I–L), or 1200 (M–P) mOsmol/kg H_2_O medium for 18 h. With an increase in osmolality, TonEBP tanslocated to the nucleus. Blue counterstain: DAPI. Scale bars: 20 μm. (Q–R) Western blot analysis of TonEBP in MDCK cells for 18 h in media of different osmolality (150–1200 mOsmol/kg H_2_O). TonEBP decreased in hypotonic conditions and increased in hypertonic conditions. Band intensity was normalized to β-actin. Each bar represents the mean of three independent experiments; error lines are SD.

**Fig 10:**
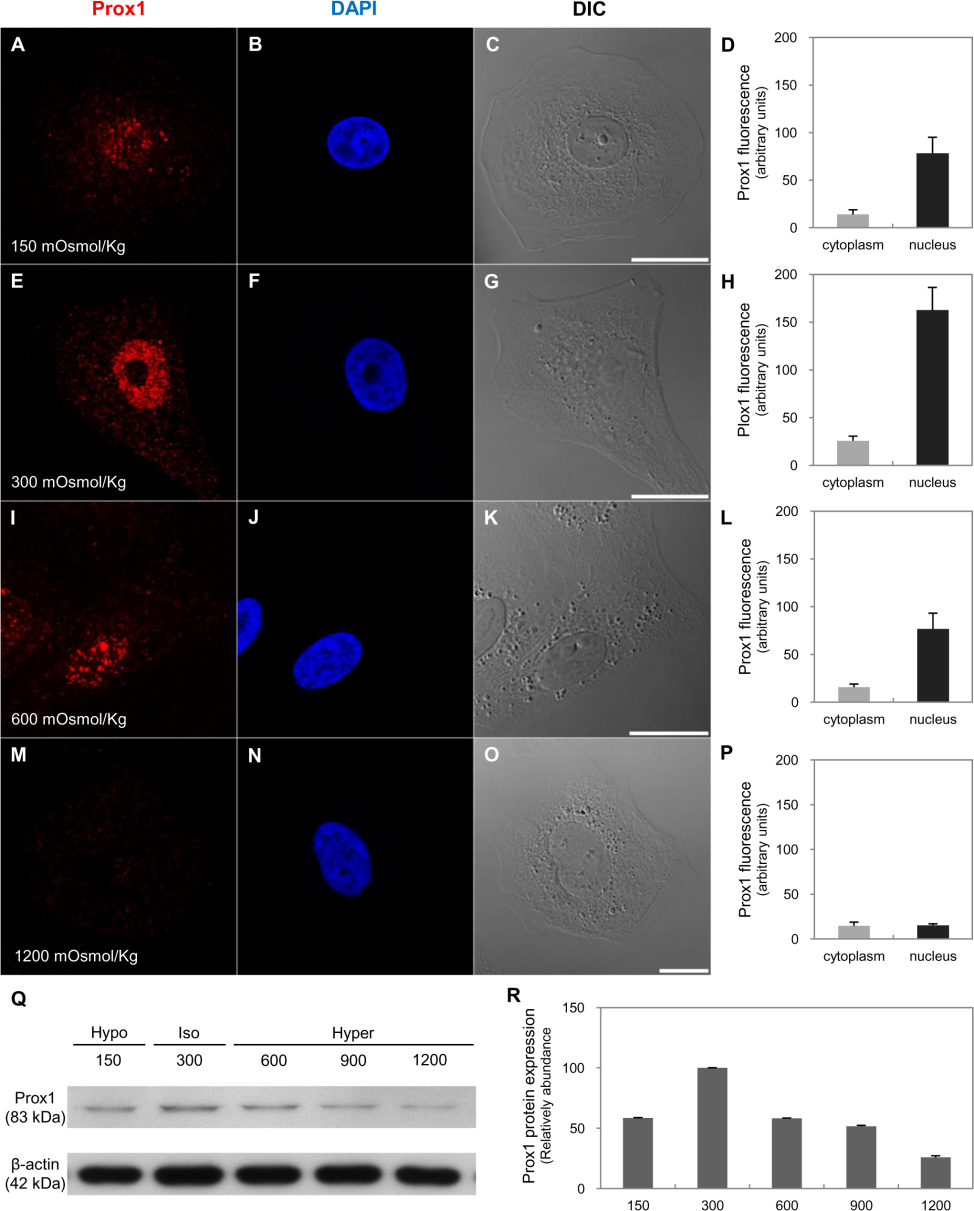
Expression and distribution of Prox1 in response to changes in osmolality *in vitro*. Immunofluorescence staining of Prox1 (red) in MDCK cells from 150 (A–D), 300 (E–H), 600 (I–L), or 1200 (M–P) mOsmol/kg H_2_O medium for 18 h. Prox1 was expressed in the nucleus and cytoplasm; nuclear intensity was strong in isotonic conditions but not in hypotonic or hypertonic conditions. Blue counterstain: DAPI. Scale bars: 20 μm. (Q–R) Western blot analysis of Prox1 in MDCK cells after 18 h in media of different osmolality (150–1200 mOsmol/kg H_2_O). Prox1 was most strongly expressed in isotonic conditions and weakened with increasing or decreasing osmolality. Band intensity was normalized to β-actin. Each bar represents the mean of three independent experiments; error lines are SD.

We used *in vitro* and *in vivo* approaches to show that Prox1 expression responds to osmolality changes. Madin-Darby canine kidney (MDCK) cells were switched to media of various osmolality (150–1500 mOsmol/kg H2O) for 18 h. With an increase in osmolality, TonEBP tanslocates to the nucleus (Fig 9A–9P). TonEBP abundance decreased in hypotonic conditions and increased in hypertonic conditions versus isotonic medium (Fig 9Q and 9R). Prox1 was expressed in the nucleus and cytoplasm; nuclear intensity was strong in isotonic conditions, but not in hypotonic and hypertonic conditions (Fig 10A–10P). Prox1 was most strongly expressed in isotonic conditions and gradually weakened with increasing or decreasing osmolality (Fig 10Q and 10R).

Initial body weight did no differ between animals. At the end of the experimental day, water-restricted animals had lost significantly more body weight than the control or water-loaded animals. Urine volume and urine osmolality changed markedly in response to changes in water intake. Urine osmolality was 2430 ± 332 mOsmol/kg H2O in control animals, but increased to 3528 ± 483 mOsmol/kg H2O in the water-restricted animals and decreased to 1043.33 ± 195 mOsmol/kg H2O in the water-loaded animals (Table 1).

**Table 1:** Water intake, body weight, and urine osmolality and volume of urine in mice maintained under different water intake conditions.

	Control (n = 6)	Water-restricted (n = 6)	Water-loaded (n = 6)
Water intake, mL/day	7.47 ± 1.46	1.89 ± 0.03^*^	19.23 ± 3.32^*^
Body weight, g	23.23 ± 0.76	17.78 ± 0.77^*^	24.40 ± 0.96
Urine volume, mL/24 h	1.98 ± 0.31	<0.50^*^	10.63 ± 2.16^*^
Urine osmolality, mOsmol/kg	2430.00 ± 332.99	3528.33 ± 483.01^*^	1043.33 ± 195.31^*^

Weight before treatment initiation was 22.44 ± 0.79. Values are means ± SD; n, no. of animals.

*P < 0.05 vs. control.

**Fig 11:**
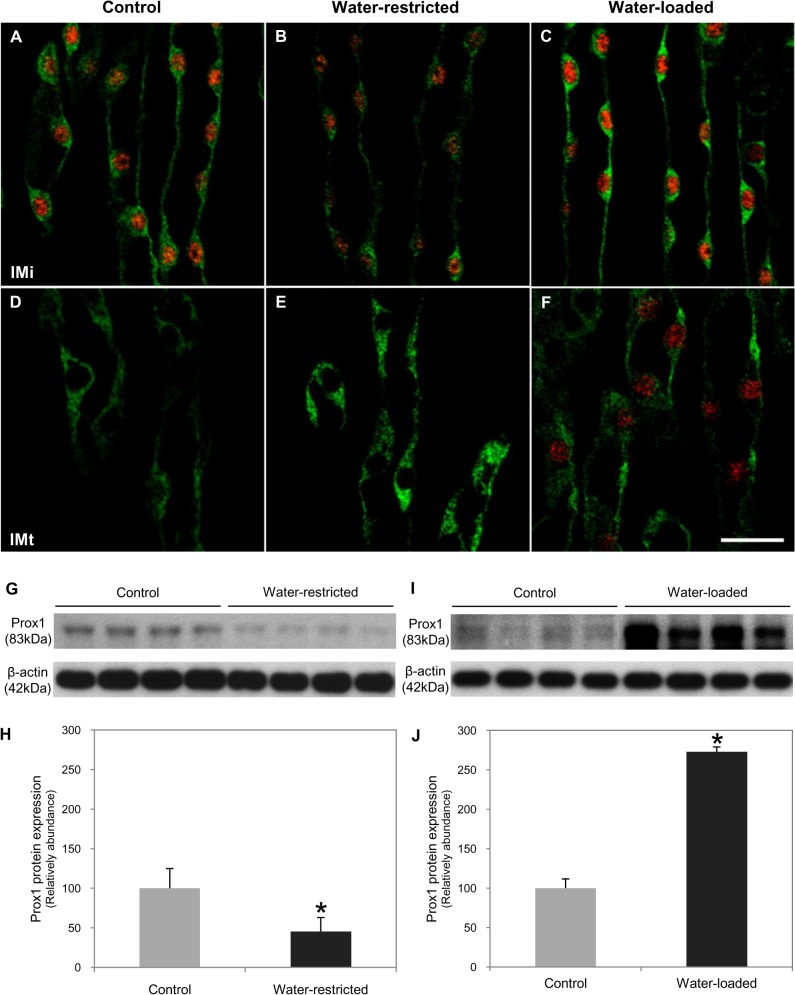
Expression and distribution of Prox1 in the renal medulla of adult mice by osmolality *in vivo*. Double immunofluorescence staining of Prox1 (red) and CLC-K1 (green) in control, water-restricted, and water-loaded mice for 7 days. In the control (A, D) and water-restricted mice (B, E), Prox1 immunoreactivity in ATL occurred only in the initial part of the renal medulla. However, Prox1 was also expressed in the terminal part of the renal medulla in water-loaded mice (C, F). Scale bars: 20 μm. (G–J) Western blot analysis of Prox1 in water-restricted or water-loaded mice after 7 days. Prox1 immunoreactivity significantly decreased in water-restricted mice and increased in water-loaded mice versus normal mice. Prox1 band intensity was normalized to β-actin. Values are means ± SD; n = 6 mice/group. *P < 0.05, experimental vs. control.

In the control and water-restricted mice, Prox1 immunoreactivity in ATL was only observed in the initial part of the inner medulla. However, Prox1 was expressed in both initial and terminal parts of the inner medulla in water-loaded mice. Prox1 protein decreased in water-restricted mice and increased in water-loaded mice compared to normal mice (Fig 11).

### Changes of Prox1 expression in the renal medulla of developing mice with reduced osmolality

**Fig 12:**
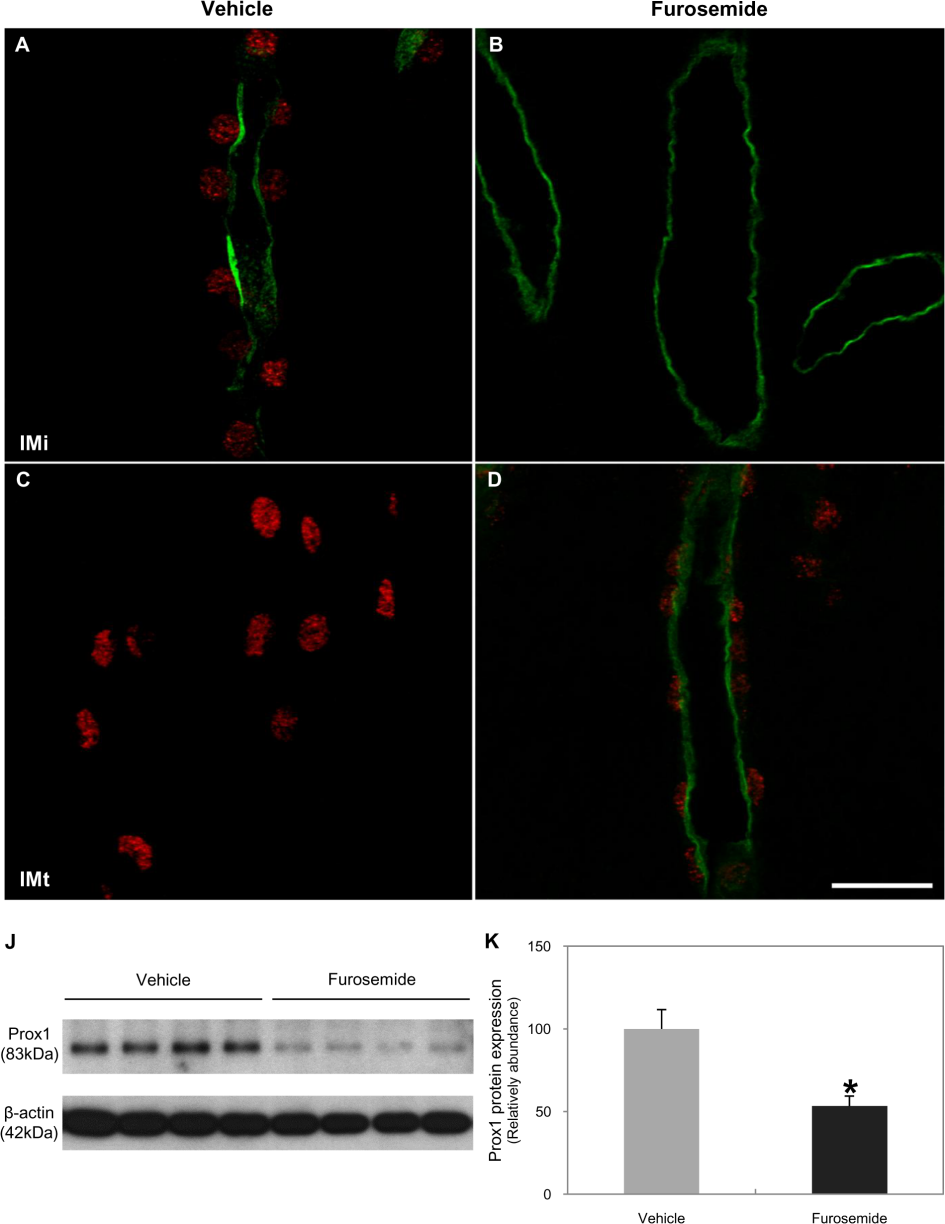
Regulation of Prox1 in the renal medulla of developing mice with reduced osmolality. Double immunofluorescence staining of Prox1 (red) and NKCC2 (green) in 7-day-old pups treated with vehicle or furosemide since birth. Prox1 was strongly expressed in the transforming NKCC2-positive TAL cells (A) and in the fully transformed NKCC2-negative ATL (C) in the vehicle group. In the furosemide-treated group, Prox1 was not expressed in the NKCC2-positive TAL cells in the base of the renal papilla due to delayed transformation of Henle’s loop (B); Prox1 was expressed in the transforming NKCC2-positive TAL cells in the tip of renal papilla (D). Scale bars: 20 μm. (J–K) Western blot analysis of Prox1 in 7-day-old pups treated with vehicle or furosemide since birth. The intensity of Prox1 immunoreactivity was significantly decreased in the furosemide-treated group vs. the vehicle group. Band intensity was normalized to β-actin. Values are means ± SD; n = 6 mice/group. *P < 0.05, experimental vs. vehicle.

The relationship between Prox1 expression and osmolality suggests that osmolality driven by NKCC2 in the developing AL induces Prox1. To test this directly, NKCC2 was inhibited in neonatal animals by daily injections of furosemide for 7 days. Urine osmolality decreased in the furosemide-treated animals, which also had lower body and kidney weights than the controls. Renal papillae were shorter in the furosemide-treated animals than in the controls (Table 2) and Prox1 expression was lower in the furosemide-treated group than in the controls (Fig 12). Transformation from the cuboidal epithelium of the thick ascending limb to a squamous epithelium in the ATL was delayed in furosemide-treated animals (S4 Fig).

**Table 2:** Effect of furosemide on body weight, kidney size, and urine osmolality.

	Vehicle (n = 6)	Furosemide (n = 6)
Body weight, g	4.305 ± 0.240	2.265 ± 0.238^*^
Kidney weight, g	0.028 ± 0.002	0.021 ± 0.001^*^
Kidney size, mm		
Length	5.590 ± 0.202	4.923 ± 0.180^*^
Width	3.048 ± 0.153	2.733 ± 0.115^*^
Thickness	2.838 ± 0.138	2.663 ± 0.109^*^
Urine osmolality, mOsmol/kg	530 ± 49.7	430 ± 14.1^*^

Values are means ± SD; n, no. of animals. Neonatal pups were injected daily with vehicle or 30 mg/kg body weight furosemide for 7 days.

*P < 0.05 vs. vehicle

## Discussion

We characterized the temporal and spatial patterns of Prox1 expression in the transforming AL of the developing mouse kidney. Prox1 was expressed first in the terminal part of TAL on F18, and then in the transforming part from TAL to ATL on P1 and in the maturing part of ATL on P4. After P7, Prox1 expression gradually disappeared from the tip in the inner papilla. In the maturing ATL, however, Prox1 expression did not completely disappear, but remained at low levels in the initial part of the inner medulla from P21 to adulthood. Expression of Prox1 in developing and adult kidneys was influenced by osmolality. These findings represent the contribution of the Prox1 in renal medullary development and epithelial differentiation that results in transformation of the epithelial phenotype in the developing AL of Henle’s loop.

The ATL is derived from the epithelium of the primitive TAL via apoptotic deletion of the TAL cells in the developing renal papilla, followed by transformation of the remaining TAL cells into the thin squamous ATL cells [3,4]. Prox1 is expressed in the cortical lymphatics and in the inner medulla of the developing mouse kidney [24].

**Fig 13:**
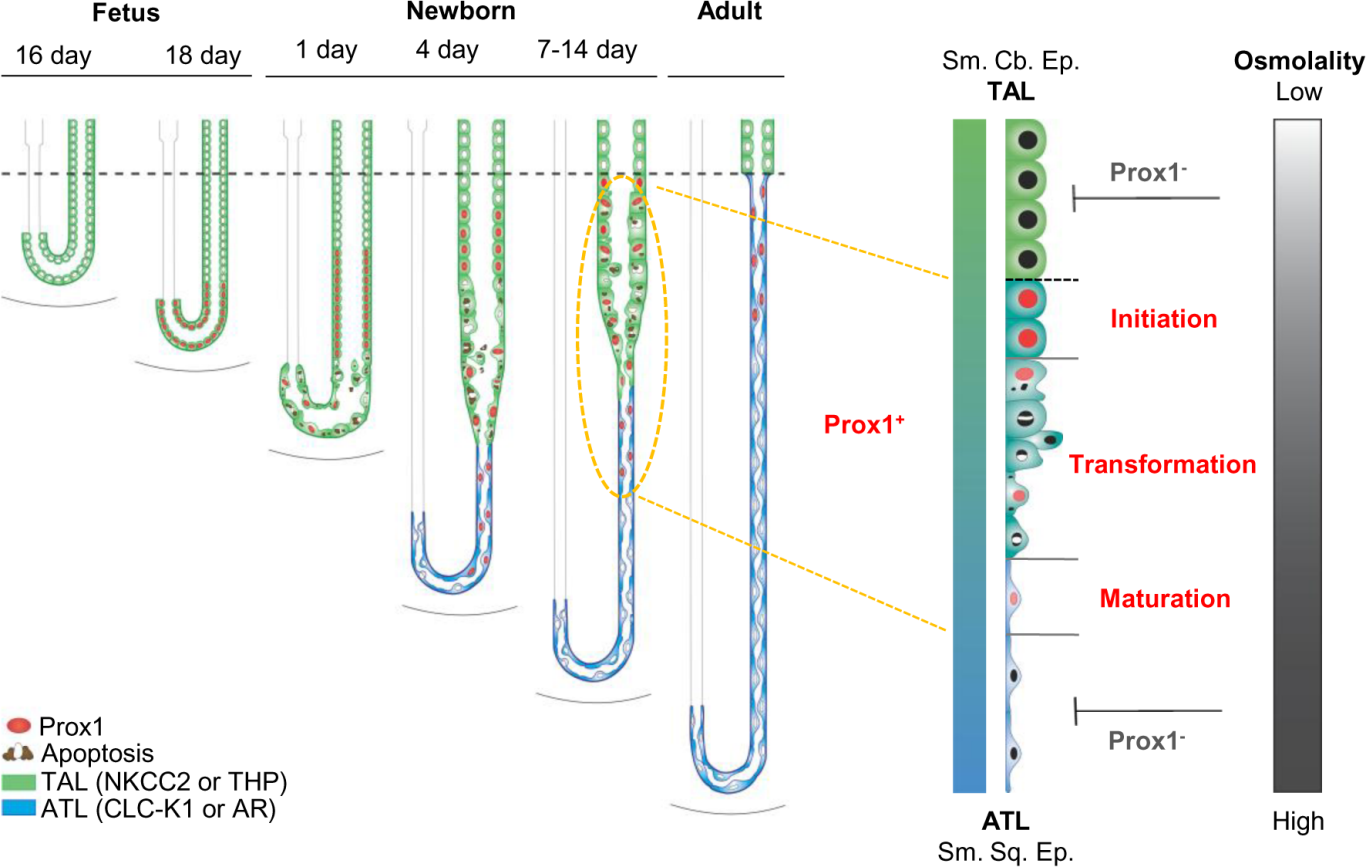
Prox1 is required for transdifferentiation of the AL of Henle’s loop in the renal medulla during mouse kidney development. Prox1 plays an important role in the maturation of the AL of Henle's loop. During the first 2 weeks after birth, some simple cuboidal epithelial cells (Sm. Cb. Ep.) of the TAL are removed by apoptosis while surviving cells are converted into the simple squamous epithelial cells (Sm. Sq. Ep.) of the ATL. Prox1 activity in TAL cells is essential for cell survival and transdifferentiation into the ATL of Henle’s loop. The location and distribution of Prox1-expressing cells is changed by increasing medullary osmolality according to the maturation of the ascending limb of Henle’s loop.

We characterized the role of Prox1 in the development of the renal medulla, especially in the transforming AL of Henle’s loop; our results are summarized in Fig 13. Prox1 expression occurred in a sequential progression through the differentiating AL of Henle’s loop. Prox1 is initially expressed in the cuboidal NKCC2-positive TAL cells in the tip of the renal papilla on F18. Prox1 expression continues in the transforming cells from P1 to P14; these transforming cells coexpressed NKCC2 or THP, a marker for TAL, and CLC-K1 or AR, a marker for ATL. Prox1 is expressed in CLC-K1- or AR-positive ATL cells from P4 until P21 and persists into adulthood. These findings suggest that Prox1 may play a role in the transforming AL of Henle’s loop.

Prox1, a prospero-related homeodomain transcription factor, is essential for the development of the lens, retina, liver, pancreas, inner ear, and lymphatic system [19,21,25]. In normal development, transdifferentiation switches a differentiated cell into another type of differentiated cell and is associated with altered gene expression. At the molecular level, differentiation is determined by expression of a master regulatory gene whose normal function is to distinguish between cell types [26–27]. There are two proposed transdifferentiation models. The natural transdifferentiation model proposes that a cell must first undergo dedifferentiation to a precursor stage before it can enter the new lineage and differentiate. The function of Prox1 is best understood in lens development. The regenerating lens of the newt perfectly illustrates the naturally occurring transdifferentiation process. In this model, Prox1 is expressed just before entering the new lineage. In the artificial transdifferentiation model, cells directly transdifferentiate into new cells, passing through an intermediate phase in which two genetic programs are active [28]. We proposed that transdifferentiation from the TAL into the ATL is an example of the artificial transdifferentiation model. First, transforming cells coexpressed NKCC2 or THP and CLC-K1 or AR. Second, Prox1 was expressed in these transforming cells as well as immediately before (NKCC2- or THP-positive cuboidal TAL) and after (CLC-K1- or AR-positive squamous ATL). Therefore, we suggest that TAL cells directly transdifferentiate to ATL cells; Prox1 might be required for the initiation and maturation of transformation.

Prox1 was not observed in TAL cells undergoing apoptosis but was expressed strongly in the remaining cells, which differentiated into CLC-K1-positive ATL epithelial cells. In dentate gyrus cell-specific Prox1-knockout mice, TUNEL-positive cells increased in number [29–30]. PCNA co-localized with a few of the Prox1-positive transforming cells. Prox1 is required for the proliferation of the lymphatic vasculature during development and is necessary for cell cycle exit and terminal differentiation [19,25,31]. Thus, Prox1 is necessary for the survival and terminal differentiation of transforming cells.

Expression of Prox1 appeared first in the transforming ALs at the tip of the renal papilla just before birth and ascended through the renal medulla during the first 2 weeks of age. The differential expression of Prox1 suggests that the process is activated by localized changes that occur around the time of birth. We hypothesized that Prox1 expression in the developing renal medulla is stimulated by osmolality. Newborn rodents, including mice, are unable to produce concentrated urine; thus, it is believed that hyperosmolality in the renal medulla is established during the first 2–3 weeks after birth [5,6,8]. The temporal sequence of expression of NKCC2, TonEBP, and its target genes during kidney development provide strong support for the hypothesis that NKCC2-driven hypertonicity is a critical signal in the functional and morphological development of the renal medulla [5–8]. During the first 2–3 weeks after birth, inner medullary NKCC2-positive TALs transform into the CLC-K1-positive ATLs in the renal medulla. Development of the CLC-K1-positive AL of Henle’s loop is concurrent with maturation of urine-concentrating abilities [2]. Our data showed that Prox1 expression increased with maturation of the AL of Henle’s loop in the developing mouse kidney, but its expression decreased with increasing urine-concentrating capacity in maturing mice. Thus, an increase in interstitial osmolality might induce Prox1 expression in the developing ascending limbs of Henle’s loop, particularly during development of the renal medulla.

To explore the relationship between osmolality in the renal medulla and Prox1 expression in the context of urine-concentrating ability, we examined Prox1 expression through various changes of osmolality *in vivo* and *in vitro*. Exposure of MDCK cells to hypertonicity (high osmolality, >600 mOsmol/kg) increased TonEBP abundance and nuclear localization [32]. In contrast, Prox1 expression increased when osmolality was closest to the range (near 300 mOsmol/kg) and decreased at the low (<150 mOsmol/kg) or high (>600 mOsmol/kg) osmolality. *In vivo*, tissue osmolality of the inner medulla from 4-day-old pups was 275±19.05 mOsmol/kg, and those of 21-day-old pups and adults were 902±47.31 mOsmol/kg and 996±15.52 mOsmol/kg respectively (data not shown). Therefore, we suggested that the optimal range of osmolality for Prox1 expression is about 300 mOsmol/kg in the developing mouse kidney. This correlates with Prox1 appearance in the transforming part of ALs when they enter the area corresponding to the future initial part of the inner medulla, located at the border between the outer medulla (low osmolality) and developing inner medulla (high osmolality). Renal hypoosmolality induced with furosemide inhibited Prox1 expression, and transformation of TAL to ATL was delayed [8]. These results suggest that Prox1 expression is required for transdifferentiation of the ALs via generation of the optimal range of osmolality.

Fully differentiated mature ATL cells maintain low levels of Prox1 only in the initial part of the inner medulla throughout adulthood. Water restriction decreased Prox1, while water loading increased Prox1 content in the initial and terminal parts of the inner medulla in water-loaded adult mice. During renal development, Prox1 is necessary and sufficient to specify the ATL cell phenotype in THP/NKCC2-positive TAL progenitors. Constant levels of Prox1 activity in the mature ATL are crucial to repress the default TAL program and maintain ATL fate. We propose that the phenotype of embryonic and postnatal ATL cells is extraordinarily plastic, and that the constant expression of Prox1 in ATL cells helps maintain their differentiated phenotype under normal conditions. Therefore, this is one of the few examples of a gene (*Prox1*) whose activity is required not only for cell type specification (ATL cells identity) but also to maintain the mature differentiated ATL cell fate.

In summary, we showed that Prox1 is critical for transdifferentiation in the maturation of the AL of Henle’s loop in the renal medulla during mouse kidney development. During the first 2 weeks after birth, simple cuboidal epithelial cells of the TAL are removed by apoptosis; surviving cells are converted into the simple squamous epithelial cells of the ATL. At that time, Prox1 activity in TAL cells is essential for cell survival and transdifferentiation into ATL of Henle’s loop. The location and distribution of Prox1-expressing cells is changed by raising medullary osmolality consistent with maturation of the AL of Henle’s loop. We conclude that Prox1 is critical for differentiation of the ATL and that its expression is regulated by osmolality.

## Materials and Methods

### Experimental animals

C57BL/6 mice were used in all experiments. All animal protocols were approved by the Institutional Animal Care and Use Committee at the Catholic University of Korea. Prenatal kidneys were obtained from F18 and postnatal kidneys were obtained from 1-, 4-, 7-, 14-, and 21-day-old pups and adult (8-week-old male) animals. Some of the neonatal pups were administered daily subcutaneous injections of furosemide (30 mg/kg body weight in 10% dimethyl sulfoxide, pH 7.0) or vehicle for 7 days (up to P7). Six animals from three separate litters were used. Adult mice were divided into three groups (n = 6/group): a control group with free access to water, a water-restricted group given a small amount of water (0.1 mL/g body weight per day) and a water-loaded group with free access to 3% sucrose water for 7 days before death. After completion of experiments, all animals were deeply anesthetized with 10% chloral hydrate (300 mg/kg, intraperitoneally), then were sacrificed to obtain experiment samples [33]. After a midline abdominal incision, the urinary tract was visualized, the bladder was punctured, and urine samples were aspirated immediately. Urine osmolality was measured with a freezing-point depression osmometer (Osmette A; Precision Systems Inc.; Natick, MA, USA).

### Cell culture and treatment

MDCK cells (CCL-34, American Type Culture Collection; Manassas, VA, USA) were grown in Eagle’s minimum essential medium supplemented with 10% bovine serum albumin, penicillin (100 U/mL), and streptomycin (100 mg/mL) and maintained at 37°C in a humidified incubator equilibrated with 95% air and 5% CO2. When the cells reached 60–80% confluence, some were switched to hypotonic medium (150 mOsmol/kg) by simple dilution of the culture medium with water or hypertonic (600–1200 mOsmol/kg) by addition of 5 M NaCl and cultured for 18 h. Others were maintained in isotonic (300 mOsmol/kg) medium. MDCK cells were grown on chamber slides and fixed in 4% paraformaldehyde in phosphate-buffered solution for 15 min before immunostaining.

### Western blotting

Western blot analysis was performed as described previously [34]. The primary antibodies were rabbit anti-Prox1 (Abcam; Cambridge, UK), rabbit anti-TonEBP [32], and rabbit anti-β-actin (Sigma; St. Louis, MO, USA). Quantification was performed by measuring signal intensity with ImageJ software (National Institutes of Health [NIH]; http://rsb.info.nih.gov/ij/).

### Immunofluorescence staining

Immunofluorescence staining was performed as described previously [24]. Briefly, 50-μm-thick vibratome or 4-μm-thick wax-embedded sections or MDCK cells were blocked and incubated with one or more primary antibodies. The following primary antibodies were used: rabbit anti-Prox1 (Millipore; Billerica, MA, USA), rabbit anti-LYVE-1 (Research Diagnostics; Flanders, NJ, USA), rabbit anti-NKCC2 (courtesy of Dr. Mark A. Knepper, NIH), goat anti-aldose reductase (AR; courtesy of Dr. Peter Kador, NIH), rabbit anti-AQP2 (Millipore), rabbit anti-CLC-K (Millipore), rabbit anti-AQP1 (Millipore), rat anti-CD31 (BD Bioscience; Franklin Lakes, NJ, USA), goat anti-THP (Mpbiomedicals; Aurora, OH, USA), mouse anti-PCNA (Dako; Carpinteria, CA, USA), and rabbit anti-TonEBP. Appropriate Alexa 488- (Invitrogen; Carlsbad, CA, USA), Cy3-, or Cy5- (Jackson ImmunoResearch Laboratories; West Grove, PA, USA) conjugated secondary antibodies were used for fluorescent detection; nuclei were stained with 4',6-diamidino-2-phenylindole (DAPI; Roche Molecular Biochemicals; Indianapolis, IN, USA). For wax-embedded sections, citrate buffer (pH 6.0) was used for antigen retrieval.

### Immunostaining-postembedding procedure

4-μm-thick wax-embedded sections were hydrated with graded ethanol and rinsed in tap water. They were then incubated for 30 min with methanolic H_2_O_2_, rinsed in tap water and treated with 0.5% Triton X-100 in PBS for 15 min. The sections then were rinsed in PBS three times for 10 min before being treated with 1% BSA for 1 h. Two of the consecutive sections were incubated with Prox1, respectively, whereas the third section was incubated in PBS overnight at 4°C. After being washed in PBS, the tissue sections were incubated for 2 h in horseradish peroxidase-conjugated donkey antimouse IgG Fab fragment or donkey antirabbit IgG Fab fragment (Jackson ImmunoResearch Laboratories) diluted 1:200 in PBS. For the detection of peroxidase, DAB was used as the chromogen. The sections were washed with distilled water, dehydrated with graded ethanol and xylene, mounted in Canada balsam and examined with a light microscope.

### Immunostaining-preembedding procedure

Sections of PLP-fixed tissue were cut transversely through the kidney using a vibratome at 50 μm and were processed for immunohistochemistry using an indirect immunoperoxidase method. All sections were washed three times in PBS containing 50 mM NH_4_Cl for 15 min. Before incubation with the primary antibodies, the sections were pretreated with a graded series of ethanol and then incubated for 4 h with PBS containing 1% bovine serum albumin (BSA), 0.05% saponin and 0.2% gelatin (solution A). The tissue sections were then incubated overnight at 4°C with antibodies directed against NKCC2 diluted in solution A. After several washes in PBS containing 0.1% BSA, 0.05% saponin and 0.2% gelatin (solution B), the tissue sections were incubated for 2 h in horseradish peroxidase-conjugated donkey antirabbit IgG FaB fragment (Jackson ImmunoResearch Laboratories, West Grove, PA, USA) diluted 1:100 in PBS containing 1% BSA. The tissues were then rinsed, first in solution B and then in 0.05 M Tris buffer (pH 7.6). To detect horseradish peroxidase, the sections were incubated in 0.1% 3,3′-diaminobenzidine (DAB) in 0.05 M Tris buffer for 5 min. Then, H_2_O_2_ was added to a final concentration of 0.01% and the incubation was continued for 10 min. The sections were washed three times with 0.05 M Tris buffer, dehydrated in a graded series of ethanol and mounted in Poly/Bed 812 resin (Polysciences, Warrington, CA). The sections were examined with a light microscope.

### TUNEL assay

Apoptotic cells were detected by a TUNEL assay with ApopTagPlus Fluorescein In Situ Apoptosis Detection Kit (Millipore) on 4-μm-thick wax-embedded sections according to manufacturer instructions. When sections were colabeled with TUNEL and Prox1 antibody, TUNEL was the initial procedure.

### Laser-scanning confocal microscopy and image analysis

Images were acquired on a Zeiss LSM510 Meta inverted confocal laser-scanning microscope with LSM 510 version 2.02 software (Carl Zeiss; Jena, Germany). Fluorescence immunoreactivity was measured in the nucleus and cytoplasm of each image with ImageJ.

### Quantification of apoptotic cells

Cell counting was performed in kidney sections from the developing mouse by using digital images (×400 magnification). Prox1-positive cells were counted in five fields each from the initial, middle, and terminal parts of the renal papilla, which were randomly selected from each of three animals in each age group. On the same section, TUNEL-positive cells were counted and expressed as a percentage of the total number of Prox1-positive cells in each field.

### Statistical analyses

All data are presented as means ± SD (*n*), where *n* indicates the number of animals studied. Differences between groups were evaluated using the Student’s t-test or one-way analysis of variance. Statistical significance was determined as *P* < 0.05.

## Supporting Information

S1 Fig Prox1 expression in NKCC2-positive thick ascending limb of developing mouse kidney. Double immunofluorescence staining for Prox1 (A, red) and NKCC2 (B, white) in renal papilla of 18-day-old fetuses. Prox1 was co-expressed in NKCC2-positive TAL in the tip of the renal papilla. Blue counterstain: DAPI. Differential intensity contrast: DIC. Scale bars: 10 μm.
(TIF)

S2 Fig Prox1 during maturation of the AL of Henle’s loop in inner medulla. Double immunostaining for Prox1 (A and C, green) and THP (B and C, red), Prox1 (D and F, green) and CLC-K1 (E and F, red) in inner medulla of 4- (A-C) and 14-day mouse kidney (D-F). Prox1 was observed in the transforming region from TAL to ATL. Prox1 expressed in the transforming immature TAL, but not in the mature TAL (A-C). Prox1 was expressed in the immature ATL, but not in the mature ATL (D-F). Scale bars = 20μm.
(TIF)

S3 Fig Cells undergoing apoptosis in the NKCC2-positive TAL of the developing mouse kidney. Differential-interfernce contrast (DIC) micrograph of the inner papilla from 18-day-old fetus (A) and 4- (B) and 7-day-old (C) pups illustrating NKCC2 immunostaining in thick ascending limb. (A) At 18-day-old fetuses, NKCC2-positive thick ascending limbs were present through the renal medulla down to the tip of the renal papilla. There were no undergoing apoptotic cells in the NKCC2-positive thick ascending limb cells in this age. (B-C) In 4- and 7-day-old pups apoptotic bodies (arrows) stained with toluidine blue are present in NKCC2-positive thick ascending limb cells undergoing transformation. Scale bars: 20 μm.
(TIF)

S4 Fig NKCC2 expression from kidneys of 7-day-old pups treated with vehicle or furosemide since birth. NKCC2-positive TALs are not seen in the renal papilla of vehicle-treated anomals (A-A’) but are clearly visible in furosemide-treated animals (B-B’). In A-B, portions are shown in higher magnification in A’-B’. Scale bars: 100 μm.
(TIF)
