# Correction: Role of Prox1 in the Transforming Ascending Thin Limb of Henle's Loop during Mouse Kidney Development

**DOI:** 10.1371/journal.pone.0139498

**Published:** 2015-09-24

**Authors:** Yu-mi Kim, Wan-Young Kim, Sun Ah Nam, A-Rum Choi, Hyang Kim, Yong-Kyun Kim, Hak-Soo Kim, Jin Kim

There are errors in the Funding section. The correct funding information is as follows: This research was supported by Basic Science Research Program through the National Research Foundation (NRF) funded by the Ministry of Science, ICT and Future Planning (NRF-2012R1A2A2A01045884) and National Research Foundation of Korea (2012R1A5A2047939). The funders had no role in study design, data collection and analysis, decision to publish, or preparation of the manuscript.
