# Supplementary figures and images for: Role of Prox1 in the Transforming Ascending Thin Limb of Henle's Loop during Mouse Kidney Development
Source: PLoS One. 2015 May 19;10(5):e0127429. doi: 10.1371/journal.pone.0127429 (PMC4438060; doi:10.1371/journal.pone.0127429)

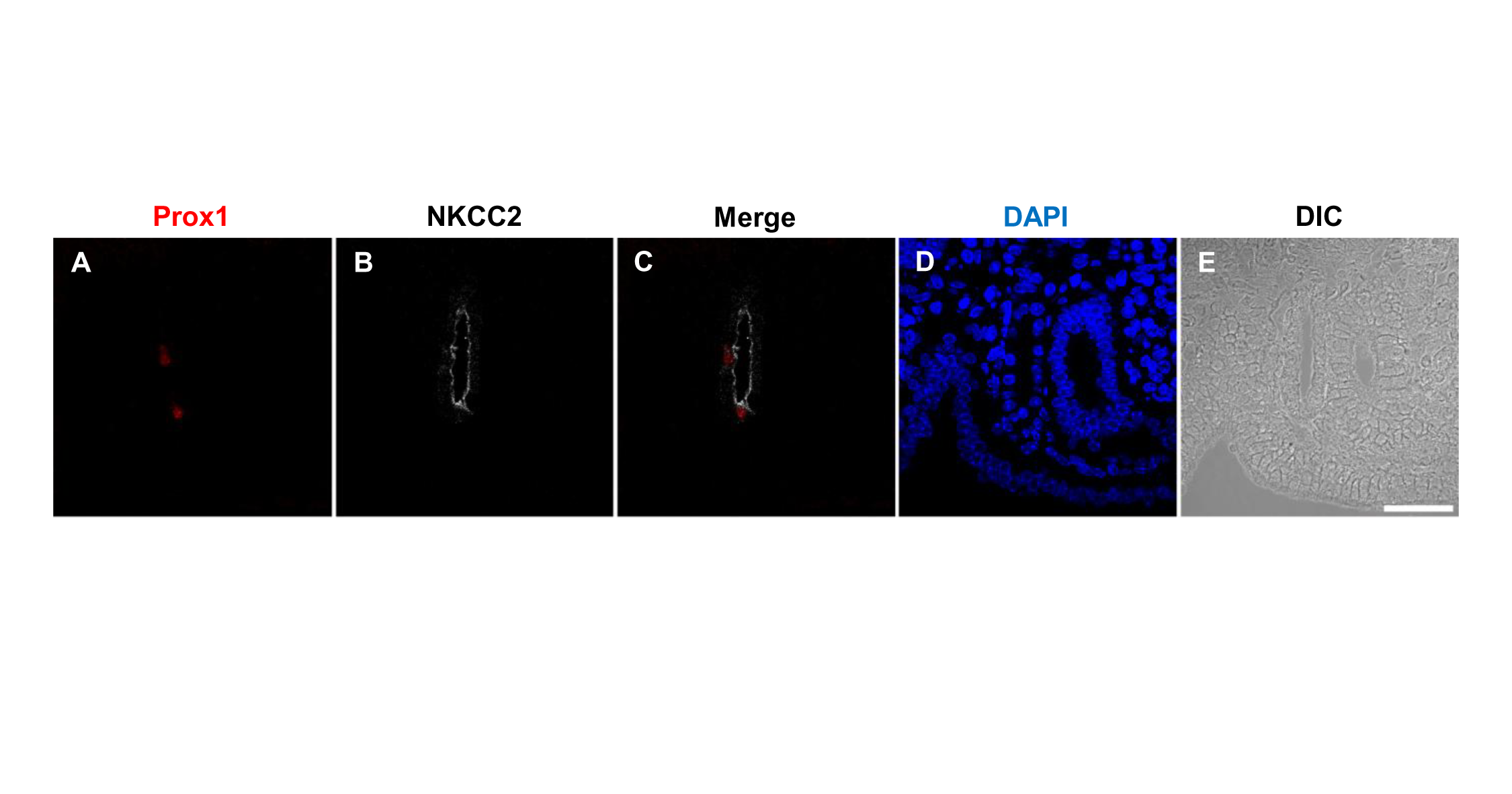

Supplement: S1 Fig — Double immunofluorescence staining for Prox1 (A, red) and NKCC2 (B, white) in renal papilla of 18-day-old fetuses. Prox1 was co-expressed in NKCC2-positive TAL in the tip of the renal papilla. Blue counterstain: DAPI. Differential intensity contrast: DIC. Scale bars: 10 μm. (TIF) [file pone.0127429.s001.tif]

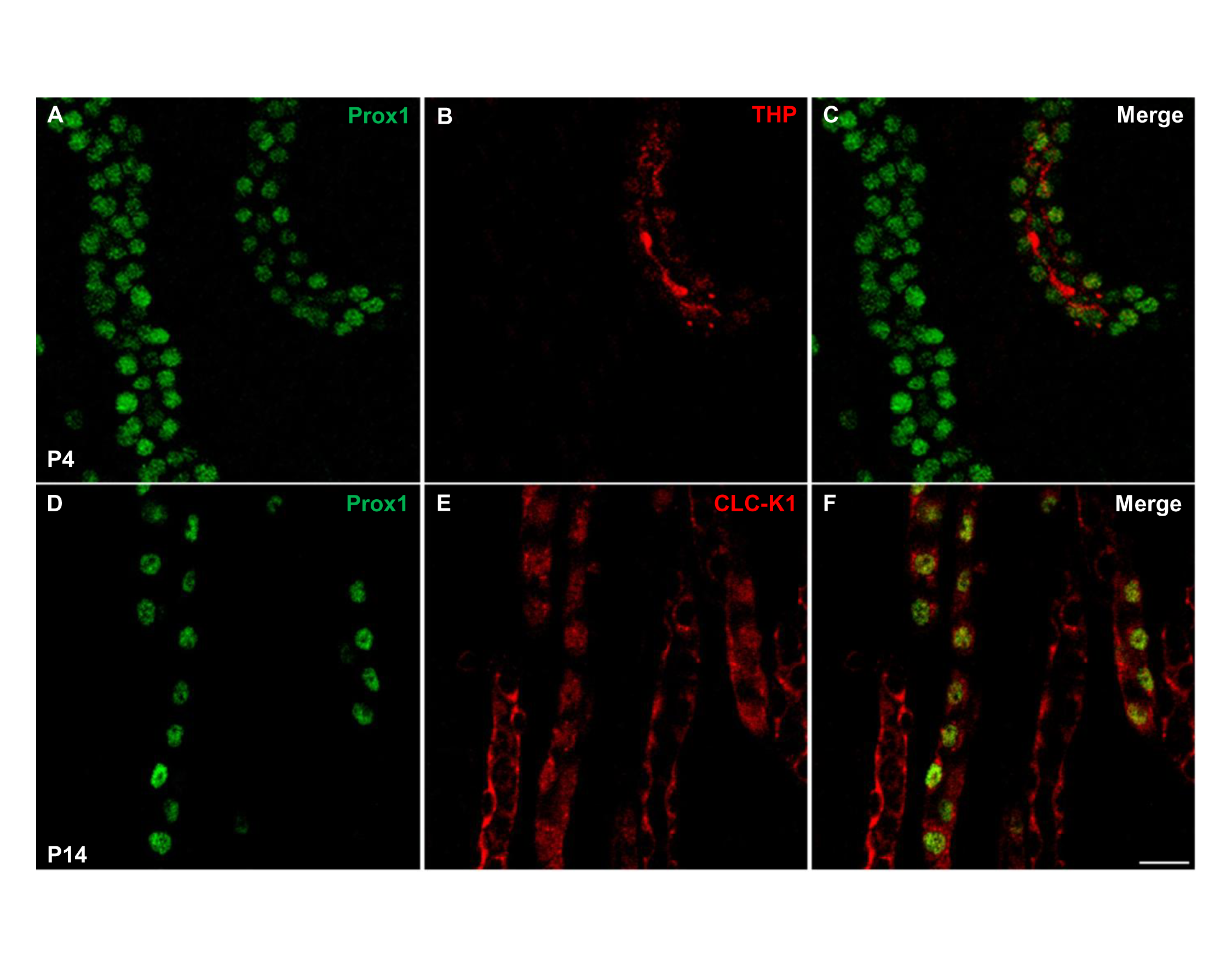

Supplement: S2 Fig — Double immunostaining for Prox1 (A and C, green) and THP (B and C, red), Prox1 (D and F, green) and CLC-K1 (E and F, red) in inner medulla of 4- (A-C) and 14-day mouse kidney (D-F). Prox1 was observed in the transforming region from TAL to ATL. Prox1 expressed in the transforming immature TAL, but not in the mature TAL (A-C). Prox1 was expressed in the immature ATL, but not in the mature ATL (D-F). Scale bars = 20μm. (TIF) [file pone.0127429.s002.tif]

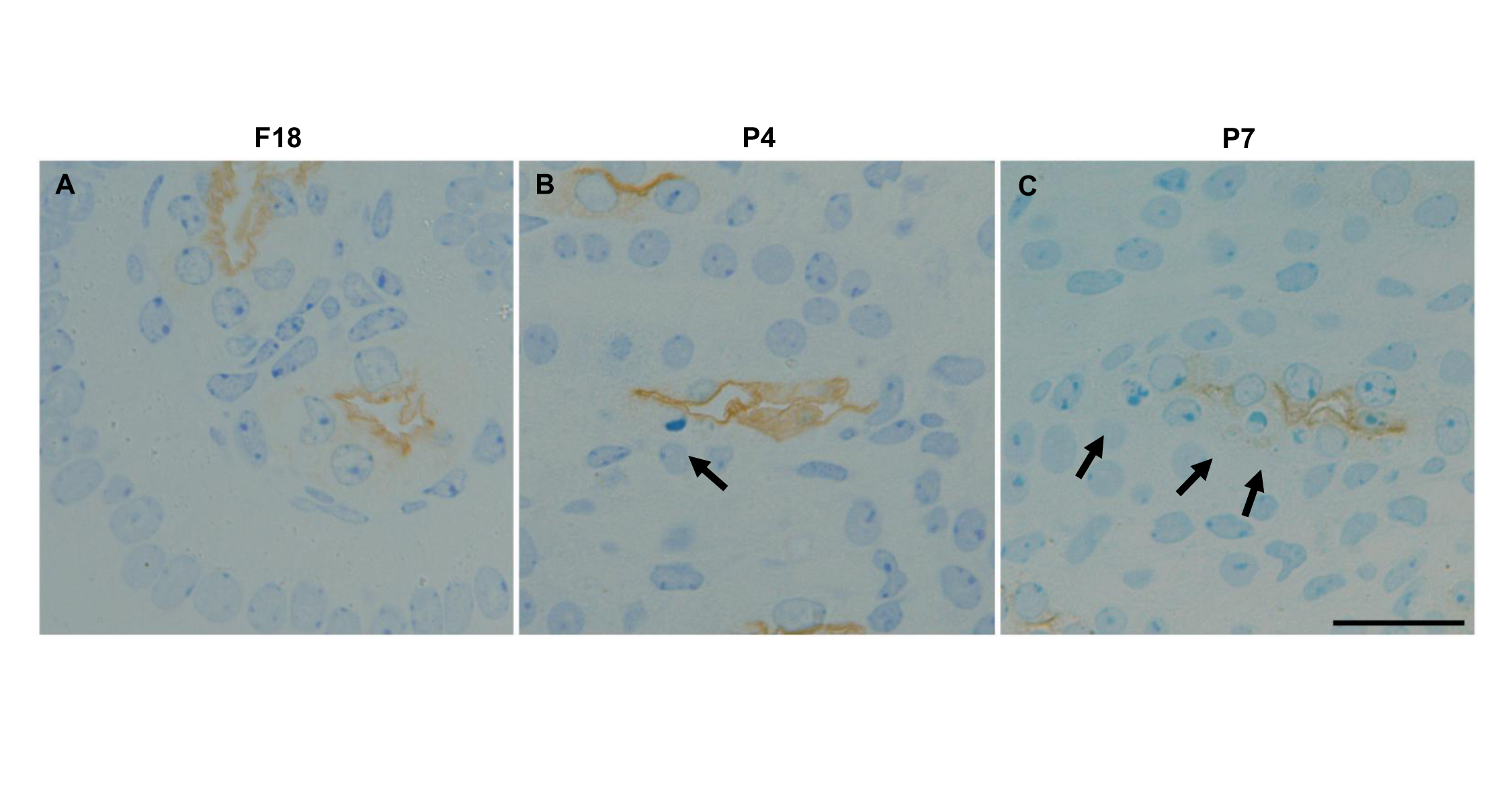

Supplement: S3 Fig — Differential-interfernce contrast (DIC) micrograph of the inner papilla from 18-day-old fetus (A) and 4- (B) and 7-day-old (C) pups illustrating NKCC2 immunostaining in thick ascending limb. (A) At 18-day-old fetuses, NKCC2-positive thick ascending limbs were present through the renal medulla down to the tip of the renal papilla. There were no undergoing apoptotic cells in the NKCC2-positive thick ascending limb cells in this age. (B-C) In 4- and 7-day-old pups apoptotic bodies (arrows) stained with toluidine blue are present in NKCC2-positive thick ascending limb cells undergoing transformation. Scale bars: 20 μm. (TIF) [file pone.0127429.s003.tif]

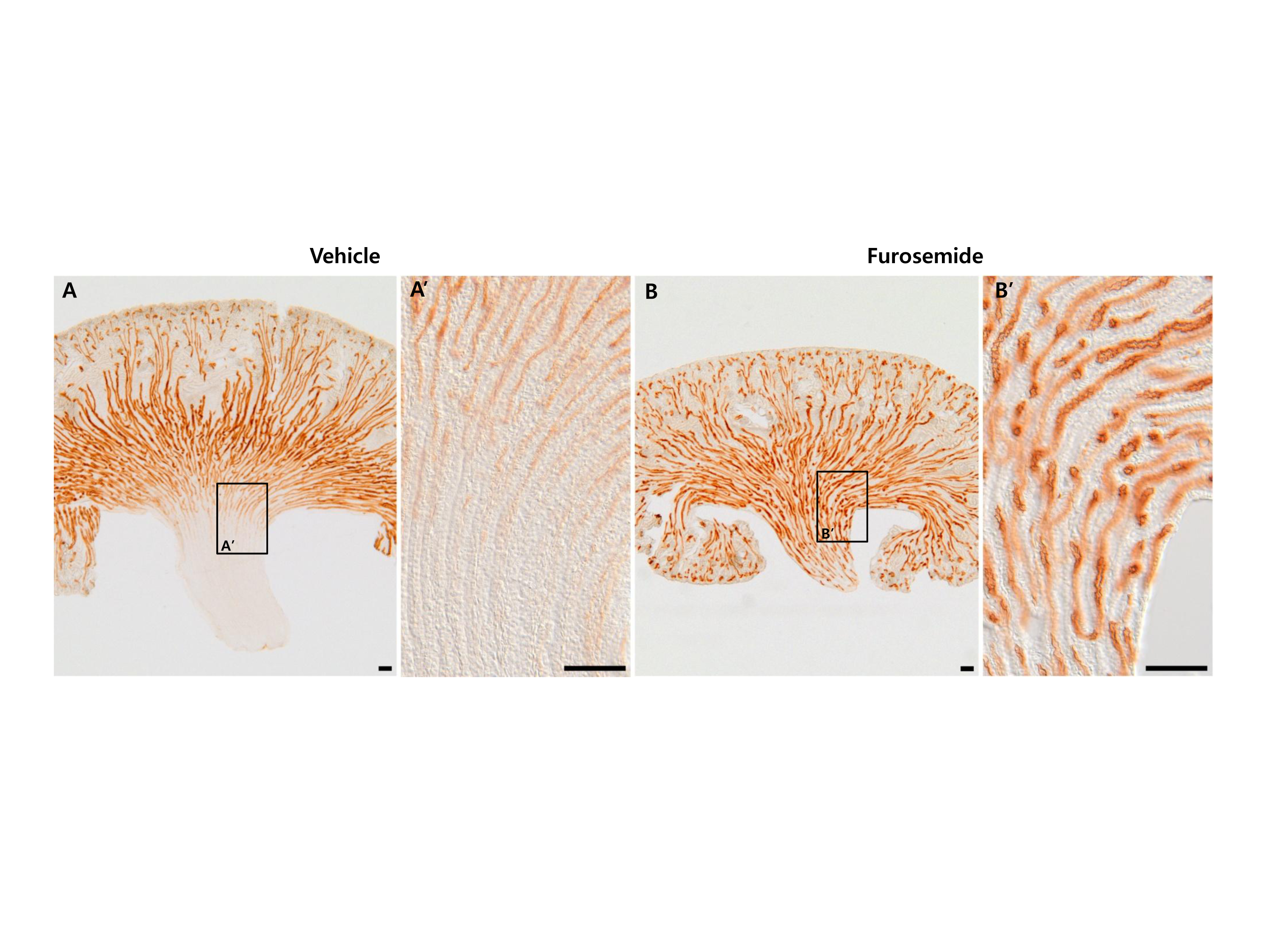

Supplement: S4 Fig — NKCC2-positive TALs are not seen in the renal papilla of vehicle-treated anomals (A-A’) but are clearly visible in furosemide-treated animals (B-B’). In A-B, portions are shown in higher magnification in A’-B’. Scale bars: 100 μm. (TIF) [file pone.0127429.s004.tif]
